# Connecting empirical phenomena and theoretical models of biological coordination across scales

**DOI:** 10.1098/rsif.2019.0360

**Published:** 2019-08-14

**Authors:** Mengsen Zhang, Christopher Beetle, J. A. Scott Kelso, Emmanuelle Tognoli

**Affiliations:** 1 Center for Complex Systems and Brain Sciences, Florida Atlantic University, Boca Raton, FL, USA; 2 Department of Physics, Florida Atlantic University, Boca Raton, FL, USA; 3 Intelligent Systems Research Centre, Ulster University, Derry∼Londonderry, Northern Ireland

**Keywords:** nonlinear dynamics, statistical mechanics, coordination dynamics, complex systems, social, complexity

## Abstract

Coordination in living systems—from cells to people—must be understood at multiple levels of description. Analyses and modelling of empirically observed patterns of biological coordination often focus either on ensemble-level statistics in large-scale systems with many components, or on detailed dynamics in small-scale systems with few components. The two approaches have proceeded largely independent of each other. To bridge this gap between levels and scales, we have recently conducted a human experiment of mid-scale social coordination specifically designed to reveal coordination at multiple levels (ensemble, subgroups and dyads) simultaneously. Based on this experiment, the present work shows that, surprisingly, a single system of equations captures key observations at all relevant levels. It also connects empirically validated models of large- and small-scale biological coordination—the Kuramoto and extended Haken–Kelso–Bunz (HKB) models—and the hallmark phenomena that each is known to capture. For example, it exhibits both multistability and metastability observed in small-scale empirical research (via the second-order coupling and symmetry breaking in extended HKB) and the growth of biological complexity as a function of scale (via the scalability of the Kuramoto model). Only by incorporating both of these features simultaneously can we reproduce the essential coordination behaviour observed in our experiment.

## 1. Introduction

Coordination is central to living systems and biological complexity at large, where the whole can be more than and different from the sum of its parts. Rhythmic coordination [1–3] is of particular interest for understanding the formation and change of spatio-temporal patterns in living systems, including, e.g. slime mould [4,5], fireflies [6,7], social groups [8,9] and the brain [10–14]. Theoretical descriptions of biological coordination are often in terms of coupled oscillators, whose behaviour is constrained by their phase relations with each other [2,15–18]. Existing studies of phase coordination often focus on systems of either very few (small-scale, mostly *N* = 2) [13,19,20], or very many oscillators (large-scale, *N* → ∞) [21–23]. Here we inquire how the two might be connected and applied to midscale systems with neither too many nor too few components. The present work answers this question by modelling empirically observed coordinative behaviour in midscale systems (*N* = 8), based on data collected in a specially designed human experiment [24]. The resultant model that captures all key experimental observations happens to also connect previous theories of small- and large-scale biological coordination in a single mathematical formulation.

But first, how are small- and large-scale models different? Small-scale models were usually developed to capture empirically observed coordination patterns, as in animal gaits [25–27], bimanual movement coordination [28,29], neuronal coordination [30], interpersonal coordination [31,32], human–animal coordination [33] and human–machine coordination [34,35]. They describe multiple stable coordination patterns (multistability) and the transitions between them (order-to-order transitions), e.g. from a trot to a gallop for a horse [36]. In humans, dyadic coordination patterns like inphase and antiphase (synchronization, syncopation) were found across neural, sensorimotor and social levels (see [13,14] for reviews), well captured by the extended Haken–Kelso–Bunz (HKB) model [29,37,38]. However, the extended HKB was restricted to describing coordination phenomena at *N* = 2 (i.e. not directly applicable to higher-dimensional coordination phenomena). By contrast, large-scale models are concerned more about statistical features like the overall level of synchrony and disorder-to-order transitions, but not so much about patterns at finer levels. As a representative, the classical Kuramoto model [2] is applicable to describing a wide range of large-scale coordination between, e.g. people [23,39], fish [40] and neural processes [22], and is often studied analytically for its incoherence-to-coherence transition (at the statistical level, for *N* → ∞; see [41,42] for reviews).

Although the extended HKB and the classical Kuramoto model emerged separately, they connect to each other by an interesting difference: the Kuramoto model with *N* = 2 is *almost* the extended HKB model except that the former lacks the term responsible for antiphase coordination in the latter (more accurately, the bistability of inphase and antiphase). Bistability of inphase and antiphase coordination, with associated order-to-order transitions and hysteresis, happens to be a key observation in small-scale human experiments [28,43]. This begs the question of whether there is a fundamental difference between large- and small-scale coordination phenomena. Does the existence of antiphase, multistability, and order-to-order transitions depend on scale *N*? With these questions in mind, we recently conducted a human experiment [24] at an intermediate scale (*N* = 8), such that the system is large enough for studying its macro-level properties, yet small enough for examining patterns at finer levels, ideal for theories and empirical data to meet at multiple levels of description. In the following sections, we demonstrate how the marriage between the two models (not either one alone) is sufficient for capturing empirical observations at multiple levels of description and we discuss its empirical and theoretical implications for biological coordination.

## 2. Results

### 2.1. Human coordination at intermediate scales

**Figure 1.**
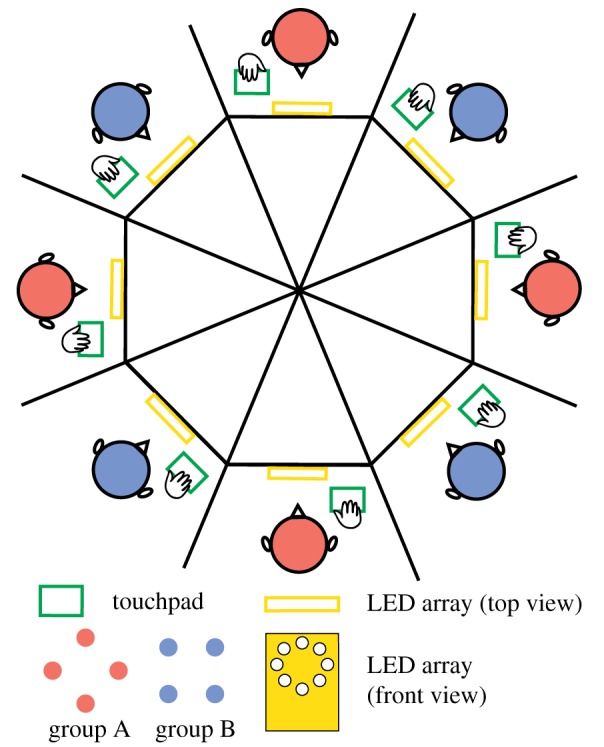
Experimental set-up for multiagent coordination. In the Human Firefly experiment [24], eight subjects interacted simultaneously with each other via a set of touch pads and LED arrays. Each subject’s movements were recorded with a dedicated touchpad. Taps of each subject were reflected as the flashes of a corresponding LED on the array presented in front of each subject. In each trial, each subject was paced with a metronome prior to interaction. The metronome assignment split the ensemble of eight into two frequency groups of four (group A and B, coloured red and blue, respectively, for illustrative purposes; the actual LEDs are all white). The frequency difference *δf* between group A and B was systematically manipulated to induce different grouping behaviour. See text and [24] for details. (Online version in colour.)

**Figure 2.**
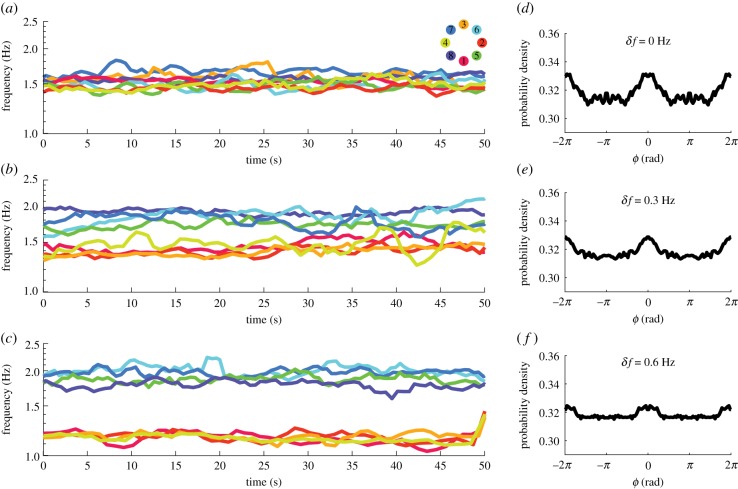
Social coordination behaviour observed in the Human Firefly experiment in terms of frequency dynamics and aggregated relative phase distributions. Panels (*a*–c) show instantaneous frequency (average over four cycles) from three example trials with diversity *δf* = 0, 0.3, 0.6 Hz, respectively. Viewed from bottom to top, in (*c*), two frequency groups of four are apparent and isolated due to high intergroup difference (low-frequency group, warm colours, paced with metronome *f*_*A*_ = 1.2 Hz; high-frequency group, cold colours, paced with metronome *f*_*B*_ = 1.8 Hz). As the two groups get closer (*b*), more cross-talk occurred between them (note contacting trajectories especially after 30 s). Finally, when the intergroup difference is gone (*a*), one supergroup of eight formed. Panels (*d*–*f*) show relative phase *ϕ* distributions aggregated from all trials for *δf* = 0, 0.3, 0.6 Hz, respectively (each distribution was computed from the set of all pair-wise relative phases at all time points in all trials for a given diversity condition; histograms computed in [0, *π*), plotted in [− 2*π*, 2*π*] to reflect the symmetry and periodicity of relative phase distributions). When diversity is low (*d*), the distribution peaks near inphase (*ϕ* = 0) and antiphase (*ϕ* = *π*), separated by a trough near *π*/2, with antiphase weaker than inphase. The two peaks are diminished as *δf* increases (*e*,*f*), but the weaker one at antiphase becomes flat first (*f*). (Online version in colour.)

Before getting into the model, we briefly review the mid-scale experiment and key results [24]. In the experiment (dubbed the ‘Human Firefly’ experiment), ensembles of eight people (*N* = 8, total 120 subjects) spontaneously coordinated rhythmic movements in an all-to-all network (via eight touchpads and eight ring-shaped arrays of eight LEDs as in figure 1; see Material and methods for details), even though they were not explicitly instructed to coordinate with each other. To induce different grouping behaviour, subjects were paced with different metronomes prior to interaction such that each ensemble was split into two frequency groups of equal size with intergroup difference *δf* = 0, 0.3 or 0.6 Hz (referred to as levels of ‘diversity’), and were asked to maintain that frequency during interaction after the metronome was turned off. Subjects’ instantaneous tapping frequencies from three example trials (figure 2*a*–*c*) show intuitively the consequences of frequency manipulations: from (*a*) to (*c*) a supergroup of eight gradually split into two frequency groups of four as diversity increased from *δf* = 0 to 0.6 Hz.

**Figure 3.**
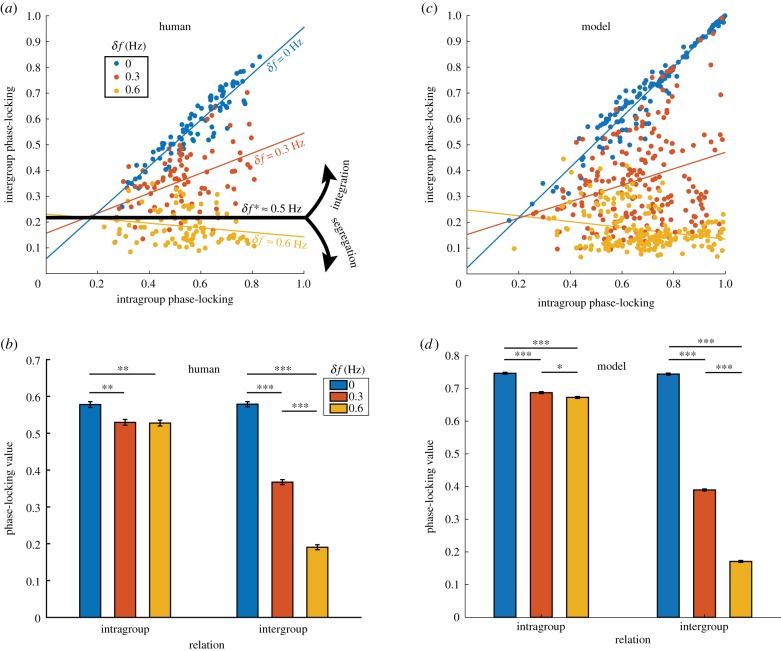
Comparison between human and model behaviour at intragroup and intergroup levels. (*a*) How intragroup coordination relates to intergroup coordination for different levels of diversity (*δf*, colour-coded) in the ‘Human Firefly’ experiment [24]. Each dot’s *x*- and *y*-coordinate reflect the level of intragroup and intergroup coordination, respectively (measured by phase-locking value; see text) for a specific trial. Lines of corresponding colours are regression lines fitted for each diversity condition (slope *β*_1_ indicates the level of integration between groups). With low and moderate diversity (blue and red), two frequency groups are integrated (positive slopes); and with high diversity (yellow), two frequency groups are segregated (negative slope). Black line (zero slope) indicates the empirically estimated critical diversity δf*, demarcating the regimes of intergroup integration and segregation. The exact same analyses applied to the simulated data (200 trials per diversity condition) and results are shown in (*c*), which highly resemble their counterparts in (*a*). (*b*) A break-down of the average level of dyadic coordination as a function of diversity (colour) and whether the dyadic relation was intragroup (left) or intergroup (right). Intragroup coordination was reduced by the presence of intergroup diversity (*δf* ≠ 0; left red, yellow bars shorter than left blue bar); intergroup coordination dropped rapidly with increasing *δf* (right three bars; error bars reflect standard errors). Results of the same analyses on simulated data are shown in (*d*), which again highly resemble those of the human data in (*b*). (Online version in colour.)

**Figure 4.**
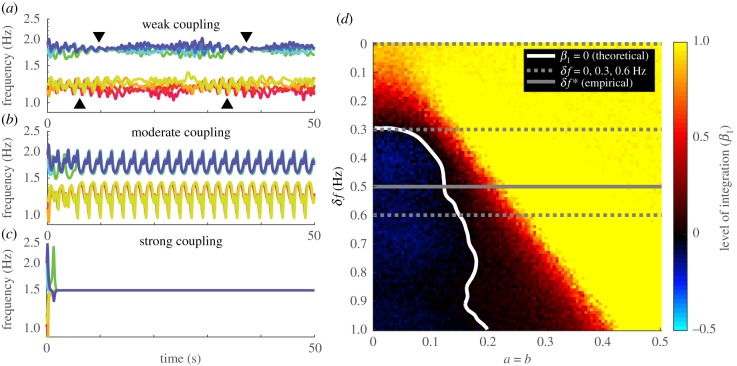
Simulated coordination dynamics changes qualitatively and quantitatively with coupling strength and frequency diversity. (*a*–*c*) Frequency dynamics of three simulated trials, with increasing coupling strength (*a* = *b* = 0.1, 0.2, 0.4, respectively) and all other parameters identical (members of the slower group, in warm colours, spread evenly within the interval 1.2 ± 0.08 Hz, similarly for members of the faster group, in cold colours, in the interval 1.8 ± 0.08 Hz; initial phases are random across oscillators but the same across trials). When the coupling is too strong (*c*), all oscillators lock to the same steady frequency. When the coupling is moderate (*b*), oscillators split into two frequency groups, phase-locked within themselves, interacting metastably with each other (dwell when trajectories are close, escape when trajectories are far apart). When the coupling is weak (*a*), intragroup coordination also becomes metastable seen as episodes of convergence (black triangles) and divergence. (*d*) Level of intergroup integration quantitatively (*β*_1_, colour of each pixel) for each combination of frequency diversity *δf* and coupling strength *a* = *b*. White curve indicates the critical boundary between segregation (blue area on the left, *β*_1_ < 0, min*β*_1_ = −0.2) and integration (red and yellow area on the right, *β*_1_ > 0). Within the regime of integration, the yellow area indicates complete integration (*β*_1_ ≈ 1) where there is a high level of phase locking, and the red area indicates partial integration (0 < *β*_1_ ≪ 1) suggesting metastability. Dashed grey lines label *δf*’s that appeared in the human experiment. Solid grey line labels the empirically estimated critical diversity. (Online version in colour.)

Key results involve multiple levels of description, in terms of intergroup, intragroup and interpersonal relations. The level of intergroup integration is defined as the relationship between intragroup and intergroup coordination (*β*_1_, slope of regression lines in figure 3*a*. Here intragroup coordination is measured by the average pair-wise phase-locking value over all intragroup dyads, and likewise, intergroup coordination over all intergroup dyads. Phase-locking value *per se* is a measure of stability of a relative phase pattern within a period of time, which equals to one minus the circular variance. See section ‘Phase-locking value and level of integration’ for technical details). Intuitively, we say that two groups are integrated if intragroup and intergroup coordination facilitate each other (positive relation between respective phase-locking values, *β*_1_ > 0), and segregated if intragroup and intergroup coordination undermine each other (negative relation between respective phase-locking values, *β*_1_ < 0. We will see later in figure 4 how this measure meaningfully captures coordination dynamics). In the experimental result, two frequency groups were integrated when diversity is low or moderate (*δf* = 0, 0.3 Hz, blue and red lines, slope *β*_1_ > 0) and segregated when diversity is high (*δf* = 0.6 Hz, yellow line, slope *β*_1_ < 0). A critical level of diversity demarcating the regime of intergroup integration and segregation was estimated to be δf* = 0.5 Hz. Within the frequency groups, coordination was also reduced by the presence of intergroup difference (figure 3*b*, left, red and yellow bars shorter than blue bar). At the interpersonal level, inphase and antiphase were preferred phase relations (inphase much stronger than antiphase; distributions in figure 2*d*–*f*), especially when the diversity was very low (figure 2*d*, peaks around *ϕ* = 0, *π*, in *radians* throughout this paper), but both were weakened by increasing diversity (figure 2*e*,*f*; in episodes of strong coordination, antiphase is greatly amplified and much more susceptible to diversity than inphase, see [24]). Notice that subjects did not remain locked into these phase relations but rather engaged and disengaged intermittently (two persons dwell near and escape from preferred phase relations recurrently, a sign of metastability [13]; see figure 6*a* red trajectory for example), reflected also as ‘kissing’ and ‘splitting’ of frequency trajectories (e.g. in figure 2*b*). In the following sections, we present a model that captures these key experimental observations at their respective levels of description.

### 2.2. A minimal experiment-based model of multiagent coordination

Our model of coordination is based on a family of *N* oscillators, each represented by a single phase angle *φ*_*i*_. We will show that a pair-wise phase coupling [2,25,29] of the form

2.1 $${\dot{\varphi }}_{i}=\omega_{i}-\sum_{\;j=1}^{N}a_{ij}\sin(\varphi_{i}-\varphi_{\;j})-\sum_{\;j=1}^{N}b_{ij}\sin2(\varphi_{i}-\varphi_{\;j}),$$

suffices to model the key features of the experimental data identified above. The left side of this equation is the time derivative of *φ*_*i*_, while the constant *ω*_*i*_ > 0 on the right is the natural (i.e. uncoupled) frequency of the *i*th oscillator. The coefficients *a*_*ij*_ > 0 and *b*_*ij*_ > 0 are parameters that govern the coupling.

Equations (2.1) include a number of well-studied models as special cases. For instance, setting *ϕ* := *φ*_1_ − *φ*_2_, *δω* := *ω*_1_ − *ω*_2_, $\tilde{a}:=a_{12}+a_{21}$ and $2\tilde{b}:=b_{12}+b_{21}$ for *N* = 2, the difference of the two resulting equations (2.1) yields the relative phase equation

2.2 $$\dot{\phi }=\delta \omega -\tilde{a}\sin\phi -2\tilde{b}\sin2\phi ,$$

of the extended HKB model [37]. The HKB model [29] was originally designed to describe the dynamics of human bimanual coordination, corresponding to equation (2.2) with *δω* = 0 (i.e. describing the coordination between two identical components). The extended HKB introduces the symmetry breaking term *δω* to capture empirically observed coordinative behaviour between asymmetric as well as symmetric components (i.e. the HKB model is included in the extended HKB model, which is further included in equations (2.1)). It has since been shown to apply to a broad variety of dyadic coordination phenomena in living systems, e.g. [13,14,19,43,44]. Equations (2.1) can be considered a generalization of the extended HKB model from 2 to *N* oscillators. It is remarkable that such a direct generalization can reproduce key features of the collective rhythmic coordination in *ensembles* of human subjects at *multiple* levels of description.

**Figure 5.**
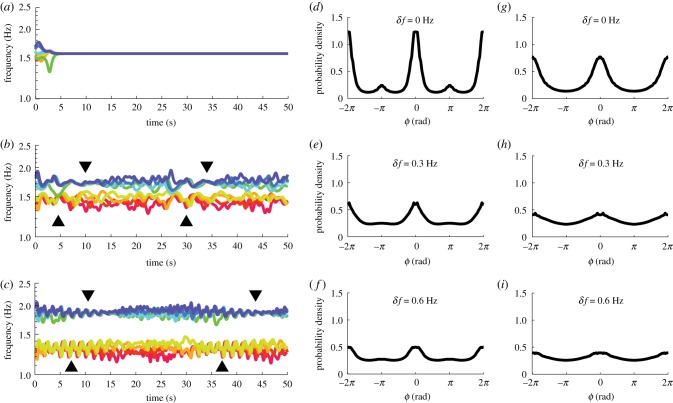
Model simulations of frequency dynamics and aggregated relative phase distributions. (*a*–*c*) An example of how intergroup difference may affect intragroup coordination using frequency dynamics of three simulated trials (*a* = *b* = 0.105; note that frequency is the time derivative of phase divided by 2*π*, and consequently the distance between two frequency trajectories reflects the rate of change of the corresponding relative phase, which increases and decreases intermittently during metastable coordination). These three trials share the same initial phases and intragroup frequency dispersion but different intergroup difference i.e. *δf* = 0, 0.3, 0.6 Hz, respectively. When intergroup differences are introduced (*b*,*c*), not only is intergroup interaction altered but intragroup coordination also loses stability and becomes metastable (within-group trajectories converge at black triangles and diverge afterwards). The timescale of metastable coordination also changes with *δf*, i.e. the inter-convergence interval is shorter for (*b*) than (*c*). (*d*–*f*) Relative phase distributions, aggregated over all time points in 200 trials (*a* = *b* = 0.105) for each diversity condition (*δf* = 0, 0.3, 0.6, respectively). At low diversity (*d*), there is a strong inphase peak and a weak antiphase peak, separated by a trough near *π*/2. Both peaks are diminished by increasing diversity (*e*,*f*). These features match qualitatively the human experiment. (*g*–*i*) The same distributions as (*d*–*f*) but for *a* = 0.154 and *b* = 0 (i.e. the classical Kuramoto model). There is a single peak in each distribution at inphase *ϕ* = 0, and a trough at antiphase *ϕ* = *π*. (Online version in colour.)

Another well-studied special case of equations (2.1) is the Kuramoto model [2], which has *b*_*ij*_ = 0 (and typically *a*_*ij*_ = *a*, independent of *i* and *j*). We will see below, however, that the Kuramoto model cannot exhibit at least one feature of the experimental data. Namely, the data show a secondary peak in the pairwise relative phase of experimental subjects at antiphase (see figure 2*d*–*f*). Simulations using the Kuramoto model do not reproduce this effect, while simulations of equations (2.1) model do (compare figure 5*d*–*f* and *g*–*i*). We give additional analytical support for this point by studying relevant fixed points of both models in the electronic supplementary materials (section ‘Multistability of the present model’).

### 2.3. Weak coupling captures human behaviour

Given the spatially symmetric set-up of the ‘Human Firefly’ experiment (all-to-all network, visual presentation at equal distance to fixation point), it is reasonable to further simplify equations (2.1) by letting *a*_*ij*_ = *a* and *b*_*ij*_ = *b* (*a*, *b* > 0),

2.3 $${\dot{\varphi }}_{i}=\omega_{i}-a\sum_{\;j=1}^{N}\sin\phi_{ij}-b\sum_{\;j=1}^{N}\sin2\phi_{ij},$$

where *ϕ*_*ij*_ = *φ*_*i*_ − *φ*_*j*_ is the relative phase between oscillators *i* and *j* (henceforth we use the notation *ϕ*_*ij*_ instead of the subtraction, since relative phase is the crucial variable for coordination [28,29]).

The behaviour of the model itself clearly depends on the coupling strength (*a*, *b*) and frequency diversity (distribution of *ω*_*i*_’s). While the latter was explicitly manipulated in the human experiment [24], the former was unknown. A qualitative look at simulated dynamics (see examples figure 4*a*–*c* for *δf* = 0.6 Hz) indicates that weak coupling better captures human behaviour (members of the same group do not collapse to a single trajectory in figure 4*a* as in figure 2). By contrast, stronger coupling (figure 4*b*,*c*) deprives the system of much of the metastability. Quantitatively, we fitted the coupling strength (assuming *a* = *b*) to the human data based on the level of intergroup integration (*β*_1_) (see distribution of model *β*_1_ in figure 4*d*) particularly for diversity condition *δf* = 0.3 Hz (i.e. using only one-third of the data to prevent overfitting). We show below how the model captures human behaviour across all diversity conditions and levels of description under the best-fit coupling strength (*a* = *b* = 0.105; see section ‘Choosing the appropriate coupling strength’ in the electronic supplementary materials for more details).

At the level of intergroup relations, model behaviour (figure 3*c*) successfully captures human behaviour (figure 3*a*) at all levels of diversity. Similar to the human experiment, low diversity (*δf* = 0 Hz) results in a high level of integration in the model (blue line in figure 3*c* slope close to 1; *β*_1_ = 0.972, *t*_199_ = 66.6, *p* < 0.001); high diversity (*δf* = 0.6 Hz) comes with segregation (yellow line slope negative; *β*_1_ = −0.113, *t*_199_ = −3.56, *p* < 0.001); and in between, moderate diversity (*δf* = 0.3 Hz) is associated with partial integration (red line positive slope far less than 1; *β*_1_ = 0.318, *t*_199_ = 4.23, *p* < 0.001). Here we did not estimate the critical diversity δf* the same way as for the human data (by linear interpolation), since we found theoretically that the level of integration depends nonlinearly on diversity *δf*, and as a result the theoretical δf* is 0.4 Hz (figure 4*d*). This prediction can be tested in future experiments by making finer divisions between *δf* = 0.3 and 0.6 Hz.

In the human experiment, not only did we uncover the effect of diversity on intergroup relations, but also, non-trivially, on intragroup coordination (outside affects within, a sign of complexity). Statistically, this is shown in figure 3*b* (three bars on the left): with the presence of intergroup difference (*δf* > 0), intragroup coordination was reduced (red, yellow bars significantly shorter than blue bar). This is well captured by the model as shown in figure 3*d* (two-way ANOVA interaction effect, *F*_2,19194_ = 3416, *p* < 0.001; the simulated data also capture the rapid decline of intergroup coordination with increasing *δf* in human data, shown in figure 3*b*,*d*, right). In addition to capturing this statistical reduction of intragroup coordination due to intergroup difference, the model, more importantly, provides a window to the dynamical mechanism underlying such statistical phenomena. For example, comparing three simulated trials with identical intragroup properties but different levels of intergroup difference (figure 5*a*–*c*), we see that the presence of intergroup difference (figure 5*b*,*c* for *δf* = 0.3, 0.6 Hz) dramatically elevates metastability in the system (compare intermittently converging–diverging dynamics in figure 5*b*,*c* to the rather constant behaviour in figure 5*a* for *δf* = 0). This suggests that the decrease of intragroup coordination in a statistical sense reflects the increase of metastability in a dynamical sense (see section ‘Examples of dynamics with intergroup coupling removed’ in the electronic supplementary materials for baseline dynamics when intergroup coupling is removed). Indeed, if we remove intragroup metastability from all simulations (by reducing intragroup frequency variability), they no longer capture the empirically observed statistical result (see section ‘Effect of reduced intragroup variability in natural frequency’ in the electronic supplementary materials).

At the interpersonal level, human subjects tended to coordinate with each other around inphase and antiphase, especially when the diversity is low (*δf* = 0 Hz; figure 2*d*, peaks around *ϕ* = 0, *π* separated by a trough near *ϕ* = *π*/2); and the preference for inphase and antiphase both diminishes as diversity increases (*δf* = 0.3, 0.6, figure 2*e*,*f* ). Both aspects are well reproduced in simulations of the model (figure 5*d*–*f*). Note that these model-based distributions are overall less dispersed than the more variable human-produced distributions (figure 2*d*–*f*), likely due to the deterministic nature of the model (i.e. no stochastic terms). Yet as demonstrated above, a deterministic model is sufficient for capturing key empirical results at all three levels of description, i.e. coexistence of inphase and antiphase tendencies and their reduction with diversity, reduction of intragroup coordination with the presence of intergroup difference, and intergroup integration∼segregation at different levels of diversity. Thus, the deterministic version of the model is preferred for simplicity.

### 2.4. The necessity of second-order coupling

Equation (2.3) becomes the classical Kuramoto model [2] when *b* = 0. We follow the same analyses as in the previous section but now for *a* = 0.154 and *b* = 0 (see section ‘Intergroup relation without second order coupling’ in the electronic supplementary material on parameter choices). The relationship between intragroup and intergroup coordination (electronic supplementary material, figure S8A; *β*_1_(0 Hz) = 0.974, *t*_199_ = 53.2, *p* < 0.001; *β*_1_(0.3 Hz) = 0.292, *t*_199_ = 4.52, *p* < 0.001; *β*_1_(0.6 Hz) = −0.011, *t*_199_ = −0.41, *p* > 0.05) resembles the case of *b* ≠ 0 (*a* = *b* = 0.105, figure 3*c*). A difference remains that for *b* = 0, *β*_1_(0.6 Hz) is not significantly less than zero (*p* = 0.68; electronic supplementary material, figure S8A yellow). The average level of intragroup and intergroup coordination also varies with diversity in the same way as the case of *b* ≠ 0 (electronic supplementary material, figure S8b for *b* = 0, interaction effect *F*_2,19194_ = 3737, *p* < 0.001, compared to figure 3*d* for *b* ≠ 0). In short, group-level statistical features can be mostly preserved without second-order coupling (i.e. *b* = 0).

However, this is no longer the case when it comes to interpersonal relations. The distributions of dyadic relative phases are shown in figure 5*g*–*i*. Without second-order coupling, the model does not show a preference for antiphase in any of the three diversity conditions, thereby missing an important feature of human social coordination (for additional comparisons between human and model behaviour, see section ‘Additional analyses on the coexistence of inphase and antiphase preference’ in the electronic supplementary materials). Analytically, we find that the coupling ratio *κ* = 2*b*/*a* determines whether antiphase is preferred (for the simple case of identical oscillators, see section ‘Multistability of the present model’ in the electronic supplementary materials). A critical coupling ratio *κ*_*c*_ = 1 demarcates the regimes of monostability (only all-inphase is stable for *κ* < 1) and multistability (any combination of inphase and antiphase is stable for *κ* > 1). This critical ratio (for equation (2.3)) is identical to the critical coupling of the HKB model [29], where the transition between monostability (inphase) and multistability (inphase and antiphase) occurs (equation (2.2); parameters in the two equations map to each other by $a=\tilde{a}/2$ and $b=\tilde{b}$). This shows how equation (2.3) is a natural *N*-dimensional generalization of the extended HKB model, in terms of multistability and order-to-order transitions.

### 2.5. The effect of non-uniform coupling

**Figure 6.**
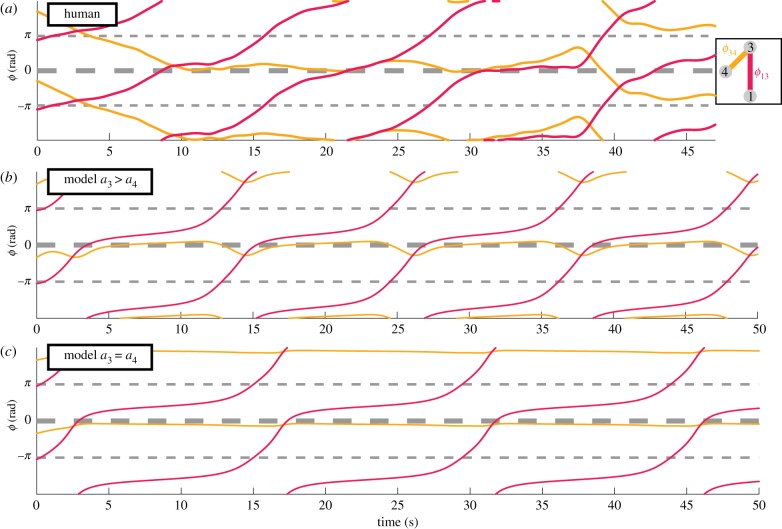
Model with non-uniform coupling captures detailed relative phase dynamics observed in human social coordination. (*a*) Experimental observation of the coordination dynamics between three persons (agent 1, 3, 4, spatially situated as in legend) in terms of two relative phases (*ϕ*_13_, *ϕ*_34_; *y*-coordinates) as a function of time (*x*-coordinates). *ϕ*_34_ (yellow) persisted at inphase for a long time (10–37 s trajectory flattened near *ϕ* = 0) before switching to antiphase (40 s; inphase and antiphase are labelled with thick and thin dashed lines, respectively, throughout this figure). *ϕ*_13_ (red) dwelt at inphase intermittently (flattening of trajectory around 10, 20, and 35 s). Three bumps appeared in *ϕ*_34_ during its long dwell at inphase (near 15, 25, 37 s), which followed the dwells in *ϕ*_13_, indicating a possible influence of *ϕ*_13_ on *ϕ*_34_. (*b*,*c*) Two simulated trials with identical initial conditions and natural frequencies, estimated from the human data. In (*b*), agent 3 is more ‘social’ than agent 4 (*a*_3_ > *a*_4_). More precisely, agent 3 has a much stronger coupling (*a*_3_ = 1) than all others (*a*_1_ = *a*_4_ = *b*_1_ = *b*_3_ = *b*_4_ = 0.105, as in previous sections). The recurring bumps in *ϕ*_34_ are nicely reproduced. In (*c*), agents 3 and 4 are equally ‘social’ (*a*_3_ = *a*_4_ = 0.5525, keeping the same average as in (*b*)). *ϕ*_34_ is virtually flat throughout the trial. (Online version in colour.)

So far, our model has captured very well experimental observations with the simple assumption of uniform coupling. However, loosening this assumption is necessary for understanding detailed dynamics. Here is an example from [24] (figure 6*a*), where coordination among three agents (1, 3 and 4, labels of locations on LED arrays) is visualized as the dynamics of two relative phases (*ϕ*_13_ red, *ϕ*_34_ yellow). Agents 3 and 4 coordinated inphase persistently (10–40 s yellow trajectory flat at *ϕ*_34_ ≈ 0), while agents 3 and 1 coordinated intermittently every time they passed by inphase (red trajectory *ϕ*_13_ becames flat, i.e. dwells, near inphase around 10, 20 and 35 s). Curiously, every dwell in *ϕ*_13_ (red) was accompanied by a little bump in *ϕ*_34_, suggesting *ϕ*_34_ was periodically influenced by *ϕ*_13_. In the framework of our model, we can approximate the dynamics of *ϕ*_34_ from equation (2.1) by assuming *ϕ*_34_ = 0 (thus *ϕ*_13_ = *ϕ*_14_),

2.4 $${\dot{\phi }}_{34}=f(\phi_{34})+\underset{=:K(\phi_{13})}{\underbrace{(a_{31}-a_{41})\sin\phi_{13}+(b_{31}-b_{41})\sin2\phi_{13}}},$$

where *f*(*ϕ*_34_) is the influence of *ϕ*_34_ on itself, *K*(*ϕ*_13_) the influence of *ϕ*_13_ on *ϕ*_34_. From *K*(*ϕ*_13_), we see that *ϕ*_13_ has no influence on *ϕ*_34_ if the coupling is completely uniform (i.e. *K*(*ϕ*_13_) ≡ 0 if *a*_31_ = *a*_41_ and *b*_31_ = *b*_41_), making it impossible to capture the empirical observation (red relation influencing yellow relation, figure 6*a*). To break the symmetry between agent 3 and 4, we ‘upgrade’ equation (2.3) to the system

2.5 $${\dot{\varphi }}_{i}=\omega_{i}-a_{i}\sum_{\;j=1}^{N}\sin\phi_{ij}-b_{i}\sum_{\;j=1}^{N}\sin2\phi_{ij},$$

where each oscillator can have its own coupling style (oscillator specific coupling strength *a*_*i*_ and *b*_*i*_). In the present case, we are interested in what happens when *a*_3_ ≠ *a*_4_ for *i* ∈ {1, 3, 4}. Two simulated trials are shown in figure 6*b* and *c* with non-uniform versus uniform coupling (same initial conditions and natural frequencies across trials, estimated from the human data). The bumps in *ϕ*_34_, accompanying dwells in *ϕ*_13_, are reproduced when *a*_3_ ≫ *a*_4_ (figure 6*b*) but not when *a*_3_ = *a*_4_ (figure 6*c*; see section ‘Additional triadic dynamics’ in the electronic supplementary materials for more analyses). This example shows that to understand interesting dynamic patterns in specific trials, non-uniform coupling strength is important.

## 3. Discussion

The present model successfully captures key features of multiagent coordination in mid-scale ensembles at multiple levels of description [24]. Similar to the HKB model [29], second-order coupling is demanded by the experimental observation of antiphase (and associated multistability) but now in eight-person coordination; and similar to the extended HKB [37], the model captures how increasing frequency difference *δf* weakens inphase and antiphase patterns, leading to segregation but now between two groups instead of two persons. This cross-scale consistency of experimental observations may be explained by the scale-invariant nature of the critical coupling ratio *κ*_*c*_ = 1, the transition point between monostability (only an all-inphase state) and multistability (states containing any number of antiphase relations). The scale invariance suggests that experimental methods and conclusions for small-scale coordination dynamics have implications for multistability, phase transitions and metastability at larger scales, and enables a unified approach to biological coordination that meshes statistical mechanics and nonlinear dynamics.

Another generalization of the classical Kuramoto model by Hong & Strogatz [45] also allows for antiphase-containing patterns (*π*-state) by letting the sign of the first-order coupling (*a*) be positive for some oscillators (the conformists) and negative for others (the contrarians). However, in contrast to our model, antiphase induced this way does not come with multistability, nor the associated order-to-order transitions observed in human rhythmic coordination [13,46]. The second-order coupling in our model allows each individual to be *both* a conformist *and* a contrarian but possibly to different degrees [47]. The simple addition of a second stable state may not seem like a big plus at *N* = 2 (2 stable states), but it rapidly expands the system’s behavioural repertoire as the system becomes larger (2^*N*−1^ stable states for *N* oscillators; with only first-order coupling, the system always has 1^*N*−1^ = 1 stable state, and therefore does not benefit from scaling up). This benefit of scale may be how micro-level multistability contributes to the functional complexity of biological systems [43,48].

Outside of the mathematical context of stability analysis, we have to recall that spontaneous social coordination is highly metastable (e.g. figure 2*a*) [24], captured by the model when frequency diversity is combined with weak coupling (e.g. figure 4*a*, in contrast to *b*,*c* under stronger coupling). Individuals did not become phase-locked in the long term, but coordinated temporarily when passing by a preferred state (inphase and antiphase) [14,43] (e.g. red trajectory in figure 6*a*). For *N* > 2, an ensemble can visit different spatial organizations sequentially (see examples in [24]), forming patterns that extend in both space and time (electronic supplementary material, figure S4 for intragroup patterns), which further expands the repertoire of coordinative behaviour (see section ‘A note on metastability’ in the electronic supplementary materials). By allowing complex patterns to be elaborated over time, metastability makes a viable mechanism for encoding complex information as real-world complex living systems do (e.g. the brain) [13,14,22,49–52]. By contrast, highly coherent patterns like collective synchronization can be less functional and even pathological [53,54]. Our results call for more attention to these not-quite coherent but empirically relevant patterns of coordination.

Besides the multistability or multi-clustering in micro patterns (a general feature endowed by higher-order coupling, e.g. [55–57]), existing mathematical studies suggest that the presence of second-order coupling should also manifest at the macro level in large-scale coordination. Naturally, second-order coupling induces multistability of the order parameter in the thermodynamic limit [58–61]. It also alters the critical scaling of macroscopic order (see [41] for a summary), i.e. for coupling strength *K* > *K*_*c*_ near *K*_*c*_, the order parameter ∥*H*∥ (norm of the order function [62]) is proportional to ${\left(K-K_{c}\right)}^{\beta }$, with *β* = 1/2 for the classical Kuramoto model and *β* = 1 when second-order coupling is added [63,64]. For complex biological systems like the brain which appears to operate near criticality [65], these two types of scaling behaviour may have very different functional implications. When modelling empirical data of biological coordination, one may want to have a closer examination or re-examination of the data for multistability and critical scaling of the order parameter, especially if finer level details are not available.

Key experimental observations are captured by our model under the assumptions of uniform coupling (everyone coordinates with others in the same way) and constant natural frequency, but these assumptions may be loosened to reflect detailed dynamics. For example, introducing individual differences in coupling style (equation (2.5)) gives more room to explain how one metastable phase relation may exert strong influence on another (figure 6*a*). Long timescale dynamics observed in the experiment (see section ‘Additional triadic dynamics’ in the electronic supplementary materials) may also be explained by frequency adaptation, which has been observed in dyadic social coordination [66]. A systematic study of the consequences of asymmetric coupling and frequency adaptation on coordination among multiple agents seems worthy of further experimental and theoretical exploration.

To conclude, we proposed a model that captured key features of human social coordination in mid-sized ensembles [24], and at the same time connects empirically validated large-scale and small-scale models of biological coordination. The model provides mechanistic explanations of the statistics and dynamics already observed, as well as a road map for future empirical exploration. As an experimental–theoretical platform for understanding biological coordination, the value of the middle scale should not be underestimated, nor the importance of examining coordination phenomena at multiple levels of description.

## 4. Material and methods

### 4.1. Methods of the human experiment

A complete description of the methods of the ‘Human Firefly’ experiment can be found in [24]. Here we provide as many details as necessary for understanding the present paper. A total of 120 subjects participated in the experiment, making up 15 independent ensembles of eight people. The protocol was approved by Florida Atlantic University Institutional Review Board and is in agreement with the Declaration of Helsinki. Informed consent was obtained from all participants prior to the experiment.

For an ensemble of eight people, each subject was equipped with a touchpad that recorded his/her tapping behaviour as a series of zeros and ones at 250 Hz (1 = touch, 0 = detach; green rectangles in figure 1), and an array of eight LEDs arranged in a ring (yellow in figure 1), each of which flashed when a particular subject tapped. For each trial, subjects were first paced with metronomes for 10 s, later interacting with each other for 50 s (instructed to maintain metronome frequency while looking at others’ taps as flashes of the LEDs). Between the pacing and interaction period, there was a 3 s transient, during which subjects tapped by themselves. Participants were instructed to match their own tapping frequency to the metronome frequency during the 10 s pacing period, and remain tapping at that frequency throughout the rest of the trial even after the metronome disappeared.

During pacing, four subjects received the same metronome (same frequency, random initial phase), and the other four another metronome. The metronome assignments created two frequency groups (say, group *A* and *B*) with intergroup difference *δf* = |*f*_*A*_ − *f*_*B*_| = 0, 0.3 or 0.6 Hz (same average (*f*_*A*_ + *f*_*B*_)/2 = 1.5 Hz). This gives rise to three conditions: (1) 1.5 Hz versus 1.5 Hz, (2) 1.65 Hz versus 1.35 Hz, and (3) 1.8 Hz versus 1.2 Hz. Each ensemble completed six trials per condition (a total of 18 trials in random order). From a single subject’s perspective, the LED array looks like the legend of figure 2*a* (all LEDs emit white light; colour-coding only for labelling locations): a subject always saw his/her own taps as the flashes of LED 1, members of his/her own frequency group LED 2–4, and members of the other group LED 5–8 (members from two groups were interleaved to preserve spatial symmetry).

From the tapping data (rectangular waves of zeros and ones), we obtained the onset of each tap, from which we calculated instantaneous frequency and phase. Instantaneous frequency is the reciprocal of the interval between two consecutive taps. Phase (*φ*) is calculated by assigning the onset of the *n*th tap phase 2*π*(*n* − 1), then interpolating the phase between onsets with a cubic spline. The relative phase between the *i*th and *j*th subject at time *t* is *ϕ*_*ij*_(*t*) = *φ*_*i*_(*t*) − *φ*_*j*_(*t*).

### 4.2. Estimating the distribution of natural frequencies

Human subjects have variable capability to match the metronome frequency and maintain it, which in turn affects how they coordinate. To reflect this kind of variability in the simulations, the oscillators’ natural frequencies were drawn from a probability distribution around the ‘metronome frequency’ (central frequencies *f*_*A*_ and *f*_*B*_ for groups *A* and *B*). To estimate this distribution from human data, we first approximated the ‘natural frequency’ of each subject in each trial with the average tapping frequency during the transient between pacing and interaction periods (see Methods of the human experiment), and subtracted from it the metronome frequency (see blue histogram in electronic supplementary material, figure S3 from the ‘Human Firefly’ experiment [24]). We then estimated the distribution non-parametrically, with a *kernel density estimator* in the form of

4.1 $$\hat{P}(x)=\frac{1}{nh}\sum_{i=1}^{n}K\left(\frac{x-x_{i}}{h}\right),$$

where the *Kernel Smoothing Function* is Normal, $K(y)=(1/\sqrt{2\pi }){\text{e}}^{-y^{2}/2}$. Here *n* = 2072 (259 trials × 8 subjects) from the experiment. We choose the bandwidth *h* = 0.0219, which is optimal for a normal density function according to [67],

4.2 $$h={\left(\frac{4}{3n}\right)}^{1/5}\sigma ,$$

where *σ* is the measure of dispersion, estimated by

4.3 $$\tilde{\sigma }=\;\frac{\text{median}\{|y_{i}-\mathrm{median}\{y_{i}\}|\}}{0.6745},$$

where *y*_*i*_’s are samples [68]. The result of the estimation is shown in electronic supplementary material, figure S3 (red curve).

### 4.3. Phase-locking value and level of integration

The (short-windowed) phase-locking value (PLV) between two oscillators (say *x* and *y*) during a trial is defined as

4.4 $${\text{PLV}}_{xy}=\frac{1}{W}\sum_{w=1}^{W}\frac{1}{M}|\sum_{m=1}^{M}\exp(\text{i}\phi_{xy}[(w-1)M+m])|,$$

where *ϕ*_*xy*_ = *φ*_*x*_ − *φ*_*y*_, *W* is the number of windows which each *ϕ* trajectory is split into, and *M* is number of samples in each window (in the present study, *W* = 16 and *M* = 750, same as [24]).

Intragroup PLV (PLV_intra_) is defined as

4.5 $${\text{PLV}}_{\mathrm{intra}}={\left(\left(\begin{array}{c} |A|\\ 2 \end{array}\right)+\left(\begin{array}{c} |B|\\ 2 \end{array}\right)\right)}^{-1}\left(\sum_{x,y\in A}\;\mathrm{PL}V_{xy}+\sum_{x,y\in B}\;\mathrm{PL}V_{xy}\right),$$

where *A* and *B* are two frequency groups of four oscillators, corresponding to the design of the ‘Human Firefly’ experiment [24], *A* = {1, 2, 3, 4}, *B* = {5, 6, 7, 8} and |*A*| = |*B*| = 4.

Intergroup PLV (PLV_inter_) is defined as

4.6 $${\text{PLV}}_{\mathrm{inter}}=\frac{1}{\left|A∥B\right|}\sum_{x\in A,y\in B}\;{\text{PLV}}_{xy}.$$

In both the human and simulated data, comparisons of PLV_intra_ and PLV_inter_ for different levels of *δf* were done using two-way ANOVA with Type III sums of squares, and Tukey honest significant difference tests for post-hoc comparisons (shown in figure 3*b*,*d*).

The level of integration between two frequency groups is defined based on the relationship between intragroup coordination (measured by PLV_intra_) and intergroup coordination (measured by PLV_inter_). The groups are said to be *integrated* if intragroup coordination is positively related to intergroup coordination, and *segregated* if negatively related. Quantitatively, for each combination of intergroup difference *δf* and coupling strength *a* (assuming *a* = *b* for our model, assuming *b* = 0 for the classical Kuramoto model), we use linear regression

4.7 $${\text{PLV}}_{\mathrm{inter},k}^{\left(\delta f,a\right)}=\beta_{0}^{\left(\delta f,a\right)}+\beta_{1}^{\left(\delta f,a\right)}\;{\text{PLV}}_{\mathrm{intra},k}^{\left(\delta f,a\right)}+\;{\text{error}}_{k}^{\left(\delta f,a\right)},$$

where ${\text{PLV}}_{\cdot ,k}^{\left(\delta f,a\right)}$ is the inter/intra-group PLV for the *k*th trial simulated with the parameter pair (*δf*, *a*), and the slope of the regression line $\beta_{1}^{\left(\delta f,a\right)}$ is defined as the measure of the *level of integration* between two frequency groups. If *β*_1_ > 0, the groups may be said to be integrated; if *β*_1_ < 0, segregated. The set $\left\{(\delta f,a)|\beta_{1}^{\left(\delta f,a\right)}=0\right\}$ is the *critical boundary* between the domains of intergroup integration and segregation. Theoretical analyses (section ‘Choosing the appropriate coupling strength’ in the electronic supplementary materials) show that this measure is meaningful (i.e. reflecting qualitative differences between dynamics; figure 4*a*–*c*).

### 4.4. Method of simulation

All simulations were done using the Runge–Kutta 4th-order integration scheme, with a fixed time step Δ*t* = 0.004 for duration *T* = 50 (matching the sampling interval and the duration of interaction period of the human experiment [24]; second may be used as unit), i.e. for system $\dot{X}=f(X)$, with initial condition *X*(0) = *X*_0_, the (*n* + 1)th sample of the numeric solution can be solved recursively

4.8 $$X[n+1]=X[n]+\frac{1}{6}(k_{1}+2k_{2}+2k_{3}+k_{4}),$$

where

4.9 $$k_{1}=\Delta t\;f(X[n]),$$

4.10 $$k_{2}=\Delta t\;f\left(X[n]+\frac{k_{1}}{2}\right),$$

4.11 $$k_{3}=\Delta t\;f\left(X[n]+\frac{k_{2}}{2}\right)$$

4.12 $$\;\text{and}\;k_{4}=\Delta t\;f(X[n]+k_{3}).$$

The solver was implemented in CUDA C++, ran on a NVIDIA graphics processing unit, solving every 200 trials in parallel for each parameter pair (*δf*, *a*). For each trial, initial phases (of eight oscillators) were drawn randomly from a uniform distribution between 0 and 2*π*, and natural frequencies from distributions defined by equation (4.2) (reflecting the design of and variability observed in the human experiment [24]). Here 200 trials are used per condition, greater than that of the human experiment (see [24] and section ‘Design of the human experiment’ in the electronic supplementary materials for details) to obtain a more accurate estimate of the mean.

## Supplementary Material

Supplementary Materials

## Data accessibility

Data of the human experiment are available online at doi:10.17605/OSF.IO/SC9P6. Simulated data are available at doi:10.17605/OSF.IO/PB7DH.
