# Supplementary material for: Connecting empirical phenomena and theoretical models of biological coordination across scales
Source: J R Soc Interface. 2019 Aug 14;16(157):20190360. doi: 10.1098/rsif.2019.0360 (PMC6731488; doi:10.1098/rsif.2019.0360)
Supplement: Supplementary Materials [file rsif20190360supp1.pdf]

Supplementary Materials

For the article "**Connecting empirical phenomena and theoretical models of biological coordination across scales**" by Mengsen Zhang, Christopher Beetle, J. A. Scott Kelso and Emmanuelle Tognoli, in the Journal of the Royal Society Interface.

Additional analyses on the coexistence of inphase and antiphase preference

The coexistence of inphase and antiphase preference in human coordination (24) (Fig 2D-F) and model behavior (Fig 5D-F) is reflected by the location of troughs (minima in the probability density functions) separating the inphase and antiphase peaks. In the human data (Fig 2D-F), the minima are at $\phi = 0.62\pi$, 0.77π, and 0.8π (away from both 0 and π) for $\delta f = 0$, 0.3, and 0.6 Hz respectively, with the minimum for $\delta f = 0$ Hz significantly less than chance ($p < 0.0005$, Fig S1A; nowhere with probability density significantly less than chance for $\delta f = 0.3$, and 0.6 Hz, as shown in Fig S1BC). This suggests that there is an unstable phase relation between inphase and antiphase, which is most prominent for $\delta f = 0$ Hz. This is well reflected in the behavior of the present model (i.e. equation (3) with $a = b = 0.105$) shown in Fig 5D-F, where the minima of the probability density functions are at $\phi = 0.6\pi$, 0.7π, and 0.67π for $\delta f = 0, 0.3$, and 0.6 Hz and the contrast between the minimum and the antiphase peak is most prominent for $\delta f = 0$ Hz (Fig 5D). On the other hand, for the Kuramoto model (i.e. equation (3) with $b = 0$), the minima of the probability density functions are always at $\phi = \pi$ for all δf's (Fig 5G-I), reflecting the instability related to antiphase when second order coupling is removed (supported by analytical results in Section Multistability of the present model). To think probablistically of how the troughs in the distributions inform us about which model better captures empirical phenomena, we consider the probability of observing the minima of three random distributions in a specific interval. The probablity of the minima of three random distributions to fall within the interval $[0.6\pi, 0.8\pi]$ (like those for the human data and simulations of the proposed model)

is 0.008, and that for interval $[0.99\pi, \pi]$ (like those for simulations of the Kuramoto model) is 10^{-6}. Thus, the convergence between human behavior and that of the proposed model, the divergence between human behavior and the Kuramoto model are highly unlikely to be due to chance. With all considered, the experimental phenomena from (24) cannot be fully captured without the second order coupling.

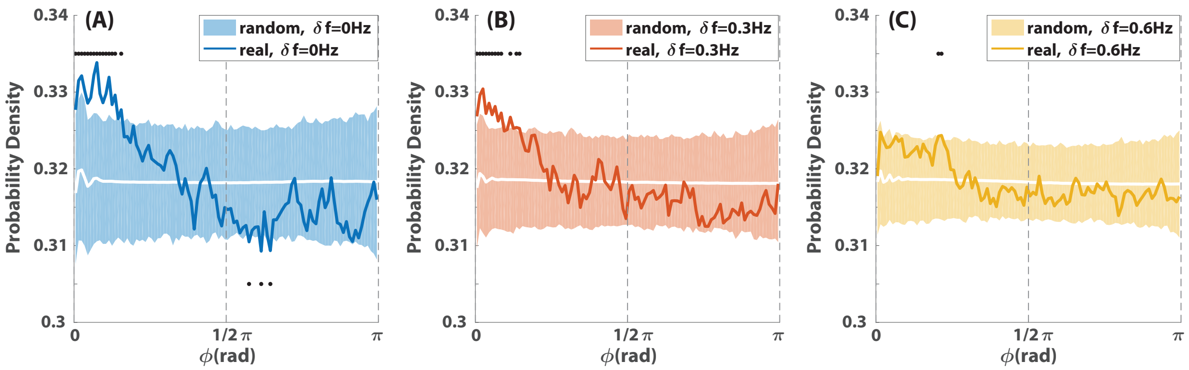

Fig S1. Relative phase distributions in the human experiment and comparisons with corresponding chance-level distributions for $\delta f = 0$ (A), 0.3 (B), and 0.6 Hz (C). coloured solid lines are the probability density functions of all dyadic relative phases for different δf's, each estimated in 100 bins. White solid lines and colour-shaded areas are the chance level distributions and corresponding confidence intervals with significance level $p = 0.0005$ per bin (after Bonferroni correction for $\hat{p} = 0.05$ for an entire distribution; see the construction of random distributions in Section D in S1 File of (24)). Black dots above the distributions mark where the probability density functions are significantly greater than chance, and black dots below mark where they are significantly less than chance (dots appear as bars when significant difference from chance is found in consecutive bins). This is a reproduction of Fig E (B1-B3) in S1 File of (24) but with all bins significantly different from chance marked (rather than as in (24), bins were marked only if significance was found in 3 or more consecutive bins).

For simulated dynamics, we show in Fig S2 that antiphase peaks in the relative phase distributions (prominent in Fig S2A, weak in Fig S2BC, which were reproduced from Fig 5D-F) did not result from transient behavior. Removal of transients from every simulated trial has little effect on the the relative phase distributions (comparing red dashed curves to black solid curves).

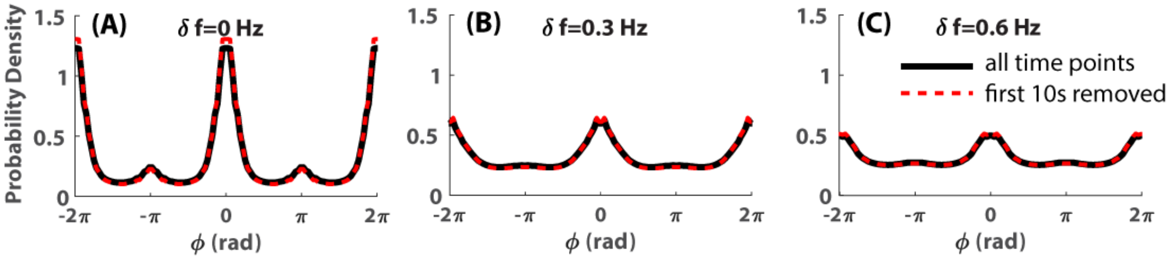

Fig S2. Aggregated relative phase distributions of simulated trials with $\delta f = 0$ (A), 0.3 (B), and 0.6 Hz (C). Each distribution was computed from 200 simulated time series. Distributions computed from all time points of all trials are plotted as black solid curves, which are identical to those in Fig 5D-F. Distributions computed from the same trials but with the first 10s of each time series discarded are plotted as red dashed curves.

Choosing the appropriate coupling strength

What we want to see is how the present model behaves as we manipulate the diversity of natural frequency ω_i's just as we did to human subjects. However, there remain two unknown parameters to be taken care of, namely the coupling strength a and b in equation (3). Before systematically finding the appropriate coupling strength, we want to first show qualitatively how it affects the dynamics.

Three simulated trials with increasing coupling strength are shown in Fig 4 from A to C, where the initial phases and natural frequencies are the same across trials (warm-color group centered around $f_A = 1.2$ Hz, cold-color group $f_B = 1.8$ Hz, corresponding to the condition $\delta f = 0.6$ Hz). When the coupling is weak ($a = b = 0.1$, Fig 4A), oscillators are well-segregated into two frequency groups. Within each frequency group, members intermittently converge (marked by black triangles) then diverge, reflecting metastability at a group level (collective dwells). For intermediate coupling ($a = b = 0.2$, Fig 4B), oscillators within each group are locked together, interacting strongly as a whole with the other frequency group (seen as the oscillation of frequency), so that the ensemble ($N = 8$) behaves like a dyad ($N = 2$). Finally, for strong coupling ($a = b = 0.4$, Fig 4C), everyone converges to a single steady frequency.

We see a progression from group-level segregation to integration from (A) to (C), indicating the important role of coupling strength in determining intergroup relation. Qualitatively, the model's behavior under weak coupling (Fig 4A) is closer to human behavior (Fig 2C) than that of stronger coupling. Next we take a more quantitative look.

To quantify the joint effect of frequency diversity (δf) and coupling strength ($a = b$ for simplicity) on integration and segregation between two frequency groups, we calculated the level of intergroup integration (β_1) for simulated trials using the same method as for the human experiment (see Phase-locking value and level of integration in Materials and Methods in the main text). For each parameter pair $(\delta f, a)$ with $a = b$, we simulated 200 trials. In each simulated trial, two frequency groups A and B each consists of four oscillators ($\varphi_1, \cdots, \varphi_4$ in group A, $\varphi_5, \cdots, \varphi_8$ in group B). The natural frequency of oscillators in group A (i.e. $\omega_1, \cdots, \omega_4$, divided by 2π) was drawn from a distribution $P(f_A)$ centered around f_A (corresponds to the metronome frequency for the group in the human experiment), and $P(f_B)$ for group B. The difference between two groups $\delta f = |f_A - f_B|$ corresponds to the level of diversity in the human experiment. Here the probability density function $P(f)$, which defines frequency dispersion within each group, was obtained by a nonparametric estimation of the empirical distribution (see Materials and Methods in the main text).

The level of intergroup integration for simulated trials is shown in Fig 4D as the colour of each pixel (diversity δf as y-coordinate; coupling strength $a = b$ as x-coordinate). Three regimes are apparent: the highly integrated (yellow, $\beta_1 \approx 1$), the partially integrated (red, $0 < \beta_1 \ll 1$), and the segregated (blue, $\beta_1 < 0$). Between the red and blue area is the critical boundary (white solid line, $\beta_1 = 0$), separating the regimes of integration and segregation. With any fixed coupling strength, for the critical boundary to fall between $\delta f = 0.3$ Hz and $\delta f = 0.6$ Hz as in the human experiment, the coupling strength has to be weak (for $\delta f = 0.6$ Hz, $\beta_1 < 0$ only when $a = b < 0.15$) but not too weak (for $\delta f = 0.3$ Hz, $\beta_1 > 0$ only when $a = b > 0.05$).

Without risking overfitting, we simply choose the coupling strength $a = b = 0.105$, for which the level of intergroup integration is the closest to experimental observation for $\delta f = 0.3$ Hz ($\beta_1 = 0.31$).

Empirical distribution of tapping frequency around metronome frequency

In the "Human Firefly" experiment (24), subjects' tapping frequency during the transient between pacing and interaction (a proxy to "natural frequency"; see Materials and Methods in main text) dispersed around the metronome frequencies. The distribution of this deviation from metronome frequencies is shown in Fig S3 (blue histogram). Most of the time, subjects were very close to the metronome frequency (peak around zero). We can use a normal distribution $\mathcal{N}(\mu, \sigma)$ to capture this peak (Fig S3 yellow line), where parameters $\mu = 0$ and $\sigma = 0.0986$ (Hz) were estimated using the median and 10th percentile of the empirical distribution. We can see a difference between the empirical distribution and the normal distribution - the normal distribution (yellow line) does not capture the fat-tails of the empirical distribution (blue bars exceed yellow line on its shoulders). These "mutant fireflies" making up the fat-tails are not to be dismissed as out-liers, because they contribute to the behavior of others in the ensemble. To better represent the empirical distribution, we used Kernel Density Estimation (with a normal kernel) as described in section Estimating the distribution of natural frequencies of Materials and Methods in the main text, and the result of estimation is shown as the red line in Fig S3 (named kernel distribution). The kernel distribution better captures the tails of the empirical distribution and was used to generate natural frequencies of oscillators in the simulations.

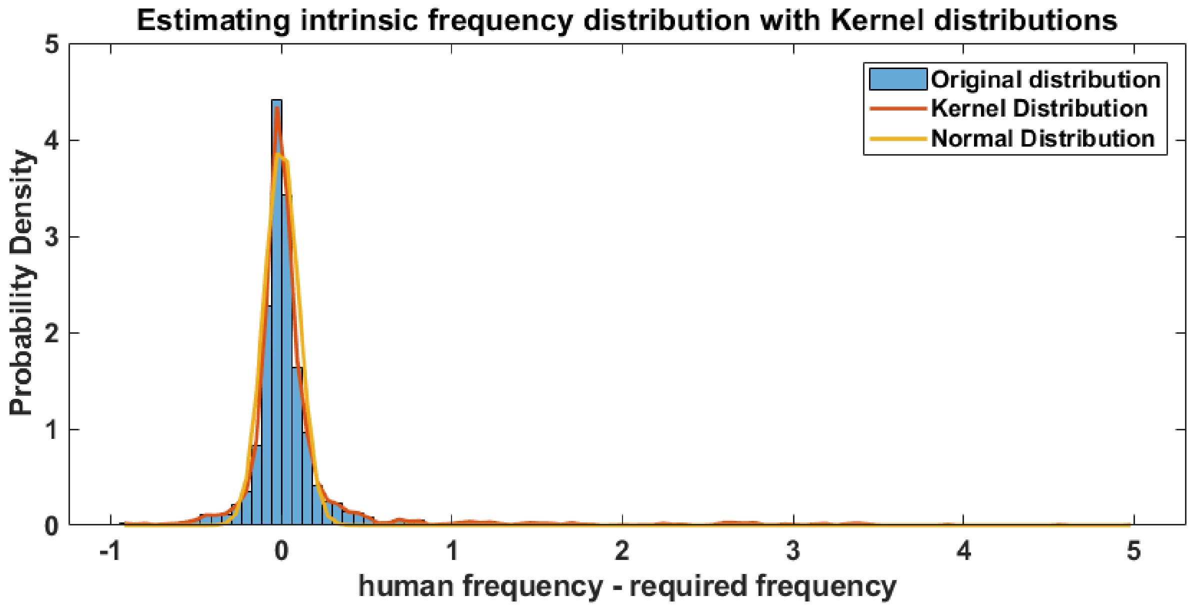

Fig S3. Distribution of human movement frequency around metronome frequencies and its estimation.

Examples of dynamics with intergroup coupling removed

By removing intergroup coupling, we obtain a modification of equation (3)

$$\dot{\varphi}_i = \omega_i - a \sum_{j=1}^{N} e_{ij} \sin \phi_{ij} - b \sum_{j=1}^{N} e_{ij} \sin 2\phi_{ij} \tag{S1}$$

where $e_{ij} = 1$ if $i, j \in \{1, 2, 3, 4\}$ or $i, j \in \{5, 6, 7, 8\}$, $e_{ij} = 0$ otherwise, for $N = 8$. The resulting dynamics (with all other parameters the same as examples in Fig 5A-C in the main text) are given in Fig S4. Within each frequency group (one group in cold colours, one group in warm colours), we see the same intragroup metastable dynamics being repeated regardless of intergroup difference ($df = 0, 0.3, 0.6$ Hz for Fig S4A, B, C respectively). These trials, without intergroup coupling, provide a baseline dynamics for comparison with Fig 5A-C, revealing the effect of intergroup influence. It turns out that for a given intragroup coupling, intragroup metastability comes from intragroup dispersion of natural frequencies. Metastability vanishes when two metastable groups have no intergroup difference (Fig 5A). In other words, without

intergroup difference ($\delta f = 0$), there are more oscillators within the same range of frequency, which cooperatively increases intragroup coordination. If we remove this intragroup dispersion of natural frequency (along with the metastability), we can no longer reproduce the experimental observation that intragroup coordination was weakened and altered by intergroup differences (see Effect of reduced intragroup variability in natural frequency for a statistical analysis).

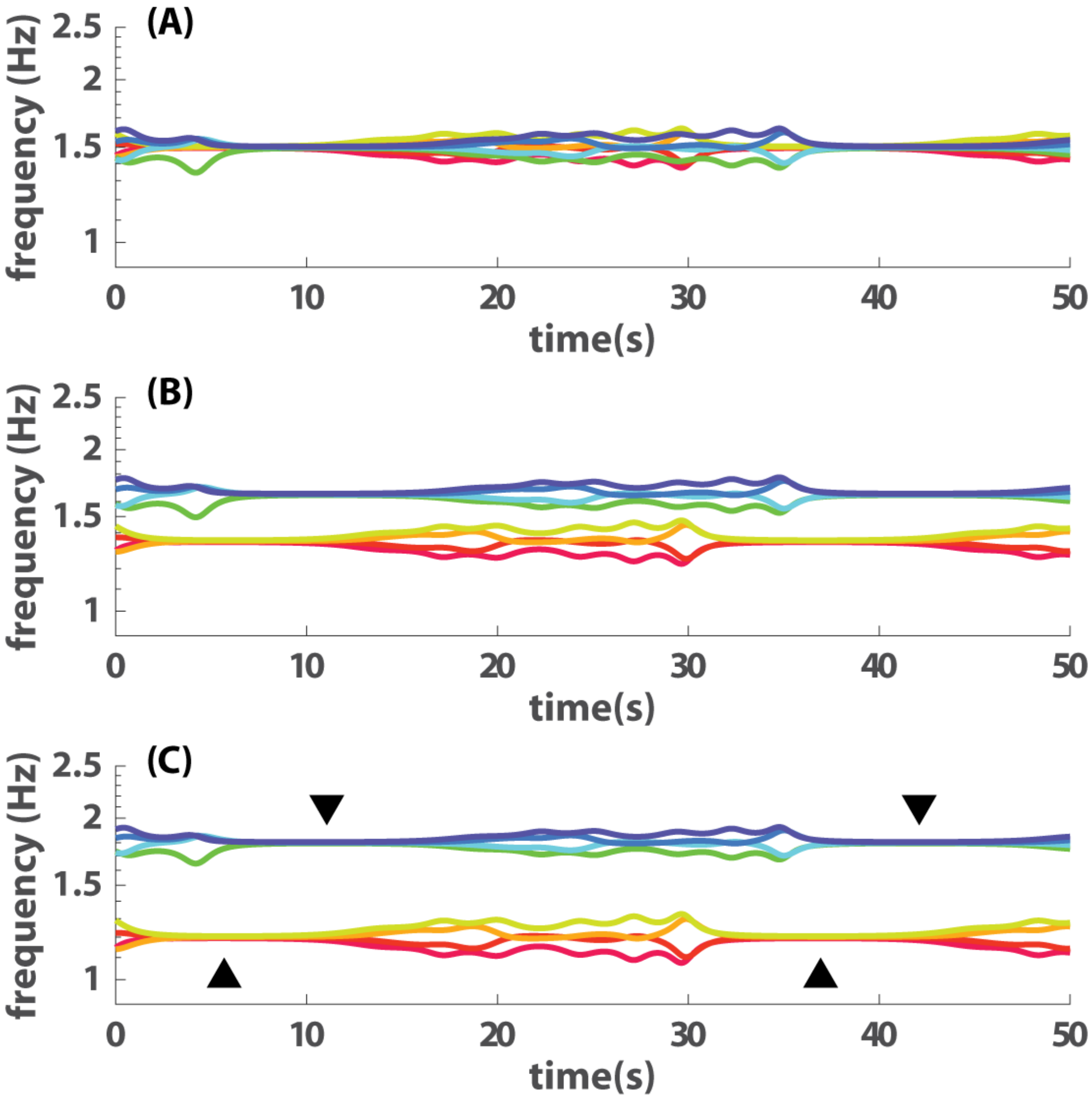

Fig S4. Intragroup dynamics without intergroup coupling, for intergroup difference $\delta f = 0$ (A), $\delta f = 0.3$ (B), and $\delta f = 0.6$ Hz (C).

With intergroup coupling, the time scale of metastability is modified by δf, as shown in Fig 5BC where the interval between two episodes of convergence (black triangles) is shorter for $\delta f = 0.3$ Hz (B) than for $\delta f = 0.6$ Hz (C). In Fig S5A, this is also visualized as the dynamics of phase-locking value (PLV) within groups (average PLV of all intragroup dyads in

3-s windows). When oscillators within groups converge, PLV is close to 1, and the interval between two consecutive peaks in a PLV trajectory reflects the time scale of the metastable coordination. Without influence from the other group, the time scales are exactly the same (trajectories exactly on top of each other in Fig S5B). With influence from the other group, the time scale depends on the level of intergroup difference (inter-peak intervals for $\delta f = 0.3$ Hz was much shorter than that of $\delta f = 0.6$ Hz in Fig S5A). Perhaps, we can consider $\delta f = 0$ Hz (i.e. lost of metastability, S5A blue line) as the special case where the inter-convergence interval is zero.

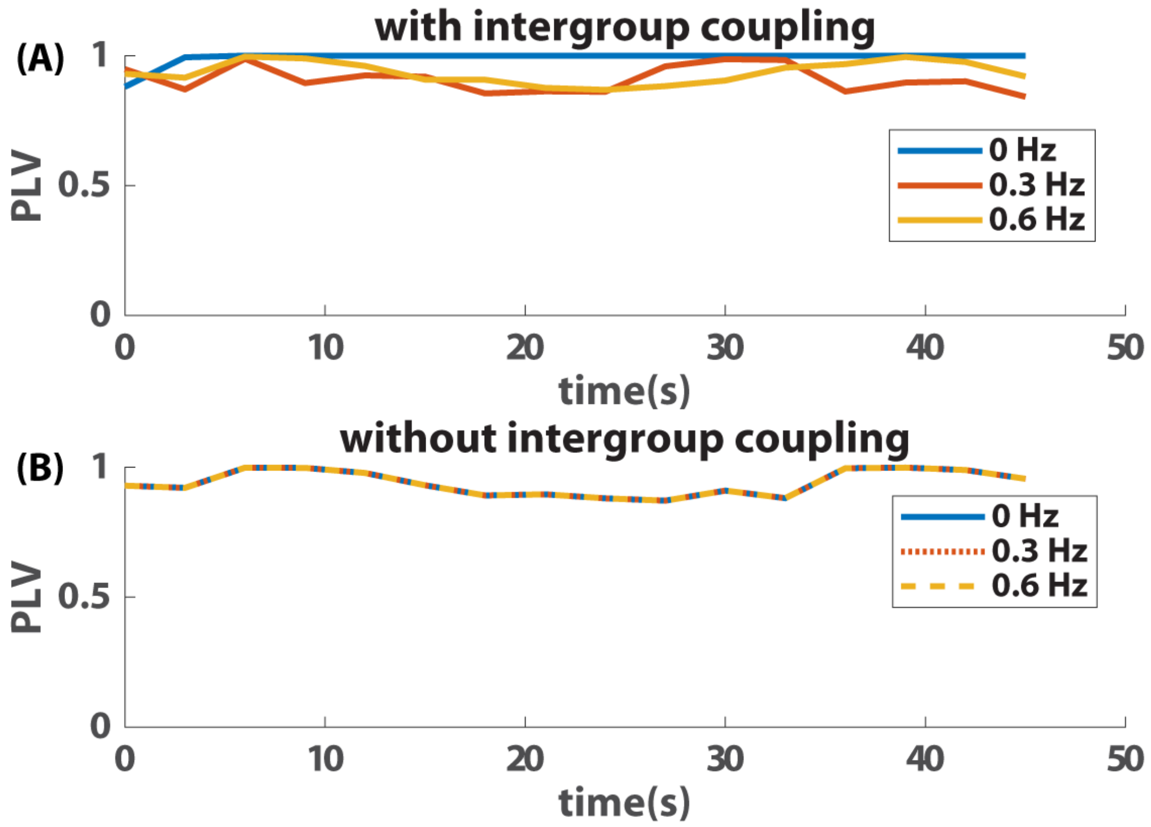

Fig S5. Dynamics of intragroup phase-locking value (PLV) with (A) and without (B) intergroup coupling.

It is also interesting to notice that for $\delta f = 0.6$ Hz, the metastable time-scale of the trial with

intergroup coupling (Fig S5A yellow line) is very similar to that of the trial without intergroup coupling (Fig S5B yellow line). This may be connected to the fact that $\delta f = 0.6$ Hz (given $a = b = 0.105$) is in the regime of intergroup segregation. It is perhaps a hypothesis worth further investigation that the level of intergroup integration (as measured by β_1, see main text) reflects how the time scale of intragroup metastability was affected by intergroup difference. Here our discussion on these examples is only to provide an intuitive understanding of the dynamics.

Effect of reduced intragroup variability in natural frequency

Recall that the reduction in intragroup coordination shown in Fig 3D (left) was based on simulations with nontrivial dispersion in natural frequency within each group, reflecting the natural variability carried into the experiment by human subjects. What if we remove that intragroup dispersion? As shown in Fig S6A (left three bars), intragroup coordination becomes all very close to the maximal level (phase-locking value close to 1) for all diversity conditions (MANOVA interaction effect $F(2, 19194) = 50152$, $p < 0.001$); we no longer see the large drop in intragroup coordination as seen in Fig 3BD. Even if we break the symmetry in coupling strength (use equation (1) with random coefficients, instead of uniform coupling in equation (3); see Random coupling for details), the phenomenon is not recovered (Fig S6B very similar to A; MANOVA interaction effect $F(2, 19194) = 59678$, $p < 0.001$). By studying the model's behavior, we found that the reduction in intragroup coordination due to intergroup difference, as observed in the human experiment, mainly depends on asymmetry in natural frequency rather than coupling strength.

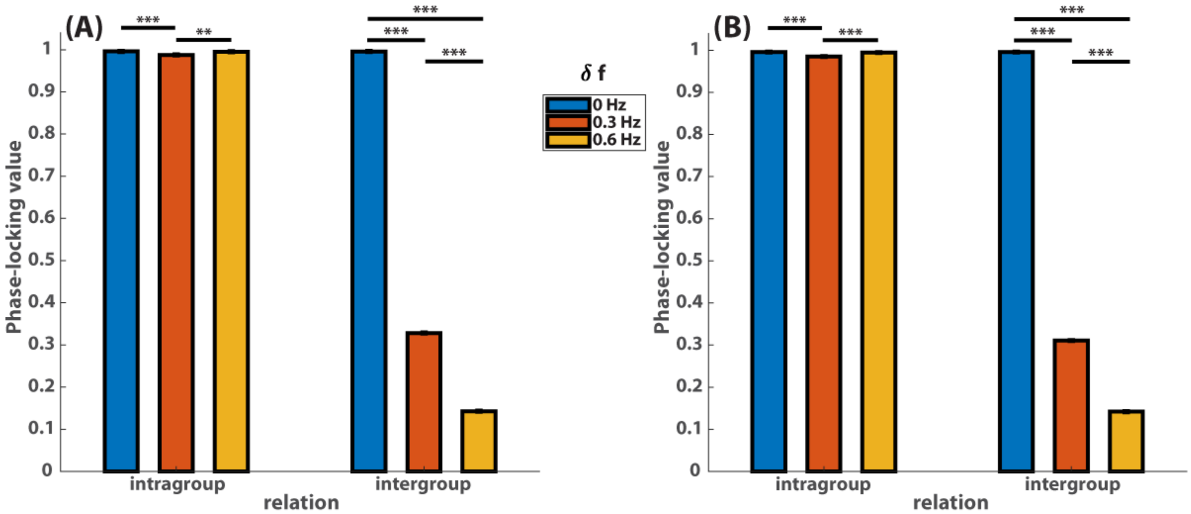

Fig S6. Intragroup and intergroup phase-locking by different levels of diversity δf for simulated data with identical natural frequency within groups. (A) shows the results of simulations with uniform coupling, and (B) non-uniform coupling (a's and b's are randomly distributed in the interval $[0, 0.2]$ see text for details).

Random coupling

To study the effect of symmetry breaking in coupling strength, we generated random coefficients for equation (1), following a uniform distribution on the interval $[0, a_{max}]$,

$$P(a) = \frac{1}{a_{max}}. \tag{S2}$$

We simulated 200 trials for each parameter pair ($\delta f = 0.3Hz, a_{max}$) for $a_{max} \in [0, 1]$ (discretized into intervals of length 0.01) with initial phases randomly distributed from 0 to 2π and natural frequencies following the empirical distribution from the human experiment (see Empirical distribution of tapping frequency around metronome frequency). We then find the value of $a_{max} = 0.2$, which produces the level of intergroup integration (β_1) closest to the experimental value (0.31). Using this fitted a_{max}, we simulated 200 trials with no intragroup dispersion in natural frequency, which were used to produce results in Fig S6B.

Intergroup relation without second order coupling

To examine whether the second order coupling term (i.e. $b \sum \sin 2\phi_{ij}$) in equation (3) is necessary for reproducing key experimental results, we let $b = 0$ and followed the exact same analysis as for the case of $b \neq 0$. The results are shown in Fig S7 (its $b \neq 0$ counterpart is Fig 4D), and Fig S8AB (its $b \neq 0$ counterpart is Fig 3CD).

Fig S7 shows the organization of the parameter space $\delta f \times a$ in terms of the level of integration between groups (β_1, see definition in main text). Similar to Fig 4D (for $b \neq 0$), the space consists of three regions - complete integration ($\beta_1 \approx 1$, yellow), partial integration ($0 < \beta_1 \ll 1$, red), and segregation ($\beta_1 < 0$, blue) - arranged from upper right to lower left. Fig S7 is approximately a scaled version of Fig 4D along a.

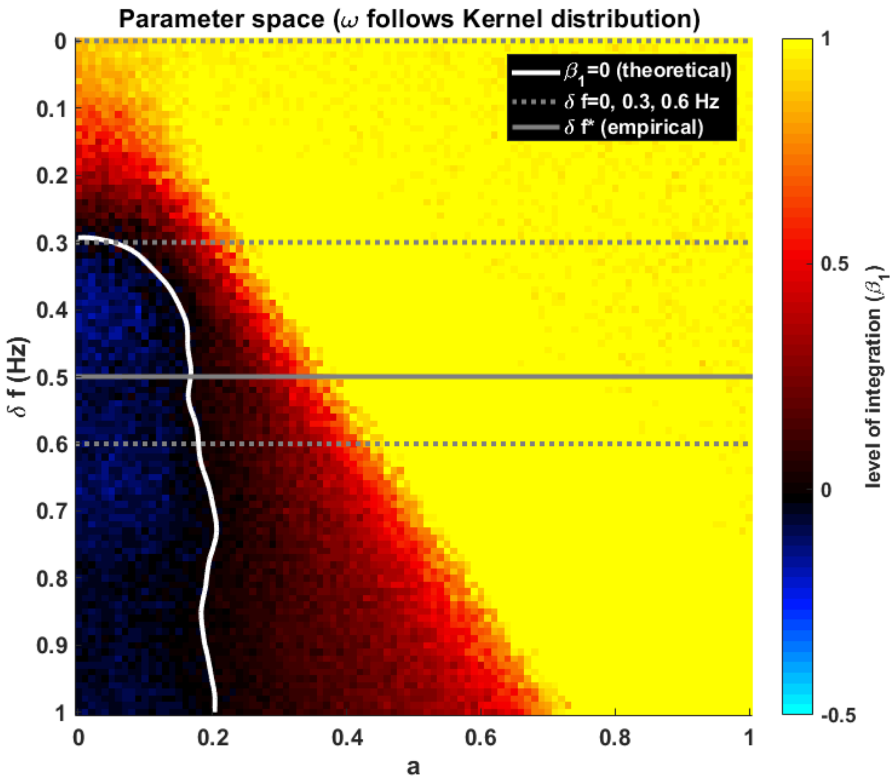

Fig S7. Level of integration between groups by δf and a, with $b = 0$.

We estimated the coupling strength to be $a = 0.154$, where the corresponding level of intergroup integration for $\delta f = 0.3$ Hz is the closest to the empirical value (up to 10^{-3} precision for a; for $a = 0.154$, $\beta_1(0.3Hz) = 0.29$, the empirical value is 0.31). The corresponding relations between intragroup and intergroup coordination is shown in Fig S8A and average intra/intergroup coordination in Fig S8B for different levels of δf.

In Fig S8A, each dot represents a particular trial with its x-coordinate indicating the average intragroup coordination (measured by phase-locking value, see Materials and Methods in main text) and y-coordinate the average intergroup coordination, whereas the colour indicates the diversity δf. Similar to the human experiment and the case of $b \neq 0$, more intragroup coordination is associated with more intergroup coordination (i.e. intergroup integration) for $\delta f = 0$ and 0.3 Hz (blue, red regression lines with positive slopes), and less intergroup coordination (i.e. intergroup segregation) for $\delta f = 0.6$ Hz (yellow regression line with negative slope). Two differences are (1) the β_1 for $\delta f = 0.6$ Hz and $b = 0$ is not significantly different from zero ($p > 0.05$; see main text for more statistics), where as its counterparts in the human data and the case of $b \neq 0$ are ($p < 0.05$); (2) in the human data and the case of $b \neq 0$, three regression lines intersect at almost the same point (see Fig 3A, C), which is not the case for $b = 0$ (Fig S8A).

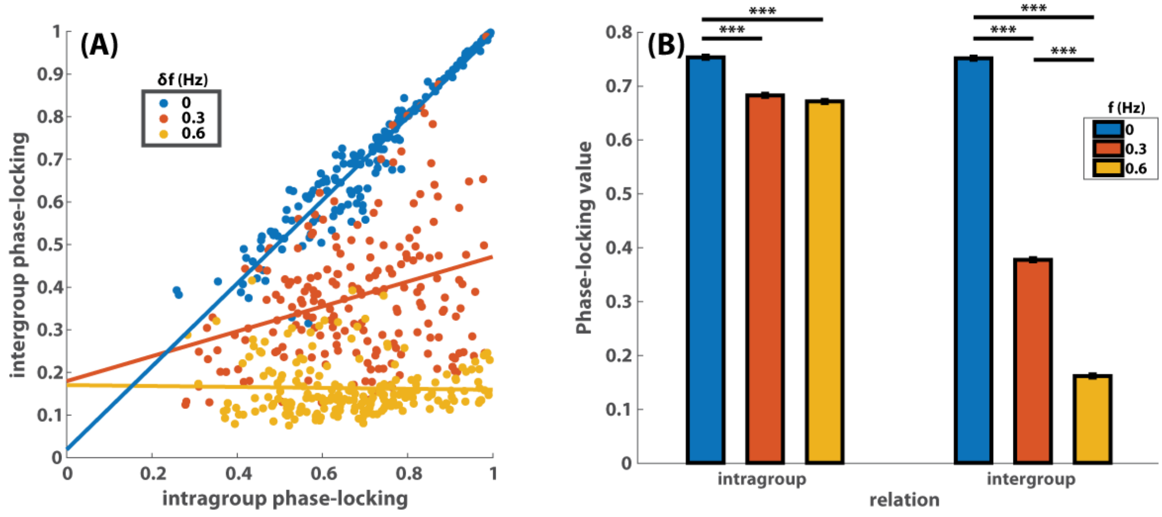

Fig S8. Intragroup, intergroup coordination and the relationship between them for $a = 0.154$ and $b = 0$. Here the level of coordination is measured by phase-locking value (see main text for definitions). (A) shows the relationship between intragroup (x-coordinate of each dot) and intergroup coordination (y-coordinate of each dot) for different levels of diversity (color code). The solid lines are corresponding regression lines whose slope quantifies the level of integration between two frequency groups. (B) shows the average intragroup (left three bars) and intergroup coordination (right three bars) for different levels of diversity (color code).

In Fig S8B, we show the average level of intragroup and intergroup coordination (again, in terms of phase-locking values). Intragroup coordination is reduced by the presence of intergroup difference (red, yellow bars on the left significantly shorter than blue bar). Intergroup coordination is more dramatically reduced by intergroup difference. Overall, these results resemble those of the human data and the case of $b \neq 0$.

Multistability of the present model

The equations for N Kuramoto oscillators with the same natural frequency, coupled to one another with a uniform coupling $a > 0$ are

$$\dot{\varphi}_i = -a \sum_j \sin(\varphi_i - \varphi_j). \tag{S3}$$

These equations can be recast in the mean-field form

$$\dot{\varphi}_i = -r\sin(\varphi_i - \psi) \qquad \text{with} \qquad r\,e^{i\psi} := a\sum_j e^{i\varphi_j}, \tag{S4}$$

which admit two types of fixed points: either **(a)** $r = 0$, or **(b)** $\sin(\varphi_i - \psi) = 0$ for each oscillator. In either case, the linearized equations governing the evolution of a small perturbation $\delta\varphi$ away from a fixed point are

$$\begin{aligned} \delta\dot{\varphi}_i &= -a\sum_j \cos(\varphi_i - \varphi_j)\,(\delta\varphi_i - \delta\varphi_j) \\ &= -r\cos(\varphi_i - \psi)\,\delta\varphi_i + a\sum_j \cos(\varphi_i - \varphi_j)\,\delta\varphi_j \end{aligned} \tag{S5}$$

We will study these linearized equations in the two cases separately.

Case (a) The first term of equation (S5) vanishes in this case. If we assume further that $\delta\varphi_j = 0$ initially for all but one oscillator, then the simplified equations are

$$\delta\dot{\varphi}_i = a\cos(\varphi_i - \varphi_j)\,\delta\varphi_j. \tag{S6}$$

In particular, $\delta\varphi_j$ itself grows exponentially at a rate a, so this fixed point cannot be stable.

Case (b) In this case there are two subgroups of oscillators, all exactly inphase within their group, and exactly antiphase to the other group. These groups cannot be equal in number because then $r = 0$ in equation (S5). Accordingly, we have $r = (n_+ - n_-)a$, where $n_+ > n_-$ are the sizes of the in- and antiphase groups (relative to the mean oscillator ψ, which of course is inphase with the larger group). The linearized equations become

$$\delta\dot{\varphi}_i = -(n_+ - n_-)as_i\,\delta\varphi_i + as_i\sum_j s_j\,\delta\varphi_j, \tag{S7}$$

where $s_i := \cos(\varphi_i - \psi) = \pm 1$ indicates whether φ_i is in- or antiphase to ψ. This equation can be recast in the matrix form

$$\delta\dot{\varphi} = J\,\delta\varphi := \left[aSHS - (a\,\mathrm{tr}\,S)S\right]\delta\varphi, \tag{S8}$$

where S is the $N \times N$ matrix with non-zero entries $s_i = \pm 1$ along the diagonal and H is the $N \times N$ matrix with all entries equal to $+1$. Our goal is to show that the Jacobian matrix J has at least one positive eigenvalue. This will imply that the fixed point is unstable dynamically. To do this, first we use the elementary identities $S^2 = 1$ and $H^2 = (\operatorname{tr} 1)H$ to calculate

$$J^2 = (a \operatorname{tr} 1)J - (a \operatorname{tr} S)\big[aHS + aSH - (a \operatorname{tr} 1)S - (a \operatorname{tr} S)1\big], \tag{S9}$$

where 1 denotes the $N \times N$ identity matrix. The additional identity $HSH = (\operatorname{tr} S)H$ then gives

$$J^3 = (a \operatorname{tr} 1)J^2 + (a \operatorname{tr} S)^2 J + (a \operatorname{tr} S)^2 \big[aH - (a \operatorname{tr} 1)1\big] \tag{S10}$$

Applying all three of these identities one last time yields

$$J^4 = (a \operatorname{tr} 1)J^3 + (a \operatorname{tr} S)^2 J^2 - (a \operatorname{tr} 1)(a \operatorname{tr} S)^2 J. \tag{S11}$$

That is, J solves a quartic polynomial, which moreover factors in the form

$$J\big[J - (a \operatorname{tr} 1)1\big]\big[J - (a \operatorname{tr} S)1\big]\big[J + (a \operatorname{tr} S)1\big] = 0. \tag{S12}$$

This is clearly the minimal-order polynomial that J solves, and it has all distinct roots. It follows that J has a complete basis of eigenvectors with eigenvalues $\lambda_0 := 0$, $\lambda_* := Na$, $\lambda_+ := (n_+ - n_-)a$, and $\lambda_- := (n_- - n_+)a$. (We can't tell the multiplicity of each of these eigenvalues from this calculation, but each has at least a one-dimensional eigenspace associated to it.) The zero eigenvalue arises because the right side of equation (S3) involves only *relative* phases, so the dynamics is insensitive to rigid rotations $\varphi_i \mapsto \varphi_i + \theta$ for all i. The eigenvalues λ_* and λ_+, meanwhile, are strictly positive, and show that this fixed point is unstable.

The lone exception to this argument occurs when $n_- = 0$, and therefore $S = 1$. Then we have $J = a[H - N1]$, which clearly has a zero eigenspace corresponding to the rigid rotation of all oscillators in the system (*i.e.*, all $\delta\varphi_i$ equal to one another). Apart from this, there is

only a single, complementary eigenspace of dimension $N-1$ associated with the eigenvalue $\lambda = -Na$. The configuration with all oscillators exactly inphase is therefore the *only* stable fixed point solution of the Kuramoto model.

Our model (with second-order coupling), on the other hand, has multiple stable fixed points for suitable values of its parameters. For uniformly coupled, identical oscillators, our equations are

$$\dot{\varphi}_i = -a\sum_j \sin(\varphi_i - \varphi_j) - b\sum_j \sin 2(\varphi_i - \varphi_j). \tag{S13}$$

The fixed points of the Kuramoto model with each φ_i equal either to ψ or to $\psi+\pi$ are also fixed points of these equations, and the linearized equations around such a fixed point are

$$\delta\dot{\varphi}_i = -(n_+ - n_-)as_i\,\delta\varphi_i + as_i\sum_j s_j\,\delta\varphi_j - 2Nb\,\delta\varphi_i + 2b\sum_j \delta\varphi_j. \tag{S14}$$

Here again we set $s_i := \cos(\varphi_i - \psi) = \pm 1$ and let $n_\pm$ denote the numbers of oscillators with $s_i = \pm 1$. The matrix form of these linearized equations is

$$\delta\dot{\varphi} = J\,\delta\varphi := \big[aSHS - (a\,\mathrm{tr}\,S)S + 2bH - (2b\,\mathrm{tr}\,1)1\big]\,\delta\varphi, \tag{S15}$$

where the matrices S, H, and 1 are defined as before. This Jacobian matrix differs from the Kuramoto Jacobian, which now we denote J_a, by its last two terms. Importantly, we have

$$J_aH = aSHSH - (a\,\mathrm{tr}\,S)H = 0 \tag{S16}$$

because $HSH = (\,\mathrm{tr}\,S)H$. It follows that all cross-terms vanish in any binomial expansion:

$$\big[J + (2b\,\mathrm{tr}\,1)1\big]^n = \big[J_a + 2bH\big]^n = J_a^n + \big[2bH\big]^n \tag{S17}$$

for all integers n. Applying these results in equation (S10) then shows that

$$\begin{aligned}\big[J + (2b\,\mathrm{tr}\,1)\big]^3 &- (a\,\mathrm{tr}\,1)\big[J + (2b\,\mathrm{tr}\,1)\big]^2 \\ &- (a\,\mathrm{tr}\,S)^2\big[J + (2b\,\mathrm{tr}\,1)\big] + (a\,\mathrm{tr}\,S)^2(a\,\mathrm{tr}\,1)1 \\ &= (a\,\mathrm{tr}\,S)^2aH + \big[2bH\big]^3 - (a\,\mathrm{tr}\,1)\big[2bH\big]^2 - (a\,\mathrm{tr}\,S)^2\big[2bH\big].\end{aligned} \tag{S18}$$

Each term on the right here vanishes if we multiply through by J. Meanwhile, the cubic polynomial on the left is the same one from the Kuramoto case, with its argument shifted by $J \mapsto J + (2b\,\mathrm{tr}\,1)1$. It factors in the same way as before to give the minimal polynomial

$$J\big[J + (2b\,\mathrm{tr}\,1 - a\,\mathrm{tr}\,1)1\big]\big[J + (2b\,\mathrm{tr}\,1 - a\,\mathrm{tr}\,S)1\big]\big[J + (2b\,\mathrm{tr}\,1 + a\,\mathrm{tr}\,S)1\big] = 0 \qquad \text{(S19)}$$

for the present model. The *non-zero* eigenvalues of the Kuramoto models are therefore all shifted by the same amount, giving $\lambda_0 = 0$, $\lambda_* = (a-2b)N$, and $\lambda_\pm := \pm(n_+ - n_-)a\,\mathrm{tr}\,S - 2Nb$. These are all negative as long as $2b > a$, the same condition that governs the HKB model for dyadic coordination. Our model is multistable when its parameters satisfy this condition.

Additional triadic dynamics

Here we provide in Fig S9 two additional variations of the simulated triadic dynamics shown in Fig 6B. Fig S9A shows what happens when all three oscillators have the identical coupling style, i.e. $a_1 = a_3 = a_4$ and $b_1 = b_3 = b_4$ (keeping the same mean coupling strength as Fig 6B and C). With the symmetry completed restored (in contrast to Fig 6C where only the symmetry between agent 3 and 4 is restored), not only the "bumps" in ϕ_{34} are gone but also the metastability altogether (at least at the observable time scale). This further illustrates the role of symmetry breaking in understanding the single-trial dynamics.

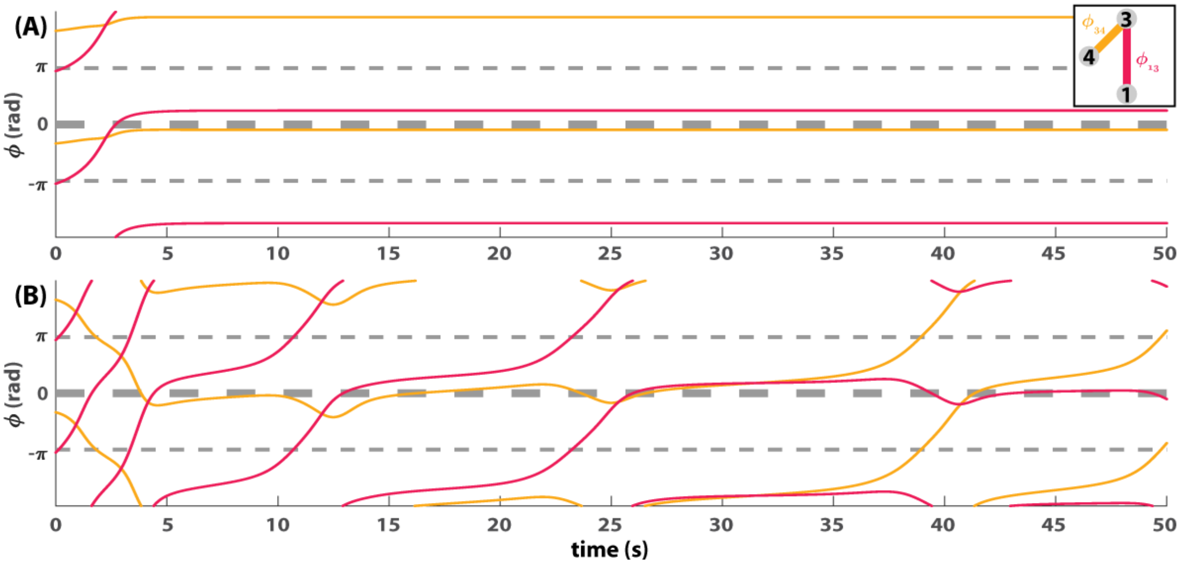

Fig S9. Simulated triadic coordination with (A) $a_1 = a_3 = a_4 = 0.4033$ and (B) varying natural frequency ω_3.

Fig S9B shows what happens when agent 3's natural frequency is not constant. A main clue suggesting a non-constant natural frequency is the increasing size of "bumps" in ϕ_{34} observed in the human behavior (see Fig 6A, the bump in yellow line at 15s was smaller than the one at 25s, and even smaller than the one at 37s) which was accompanied by growing length of the dwells in ϕ_{13} (red trajectory in Fig 6A has three periods of flattening, each one longer than the previous one). This could simply mean that agent 3's "natural frequency" was moving towards agent 1's and away from agent 4's. In the model, the natural frequencies of agent 1 and 4 are 1.57 and 1.45 Hz respectively. We simply let ω_3 increase linearly from 1.2 Hz to 1.7 Hz, instead of being constant (i.e. 1.375 Hz for Fig 6BC and S9A), over the course of the trial. The resulted dynamics is shown in Fig S9B. We see the dwells of ϕ_{13} (red line flattening around 7, 17 and 32s) are getting longer over time as the bumps in ϕ_{34} (yellow line) grow (the last bump grows out of itself at 37s and leaves inphase). In fact, at the end of the last dwell (around 37s) ϕ_{13} is no longer metastable in the original sense but begins to oscillate around inphase $\phi = 0$, whereas ϕ_{34} takes its place at that time and becomes metastable (i.e. after 37s yellow line starts

wrapping).

Gradually increasing natural frequency of agent 3 (ω_3) creates two subtle effects in addition to the increasing bump size. The first has already been hinted at that a gradual change of parameter can cause ϕ_{34} to suddenly leave inphase ($\sim$ 37s yellow line in Fig S9B). In the human trial (Fig 6A), ϕ_{34} had also, after the third bump, left inphase (37s). The difference is that the humans left for antiphase, instead of becoming metastable as for our simple model assuming linearly increasing natural frequency. This suggests that there was, unsurprisingly, more interesting adaptation going on in human movement frequency than just a linear ramping. Another subtle effect is of the same flavor but is concerned with what happens before ϕ_{34} began to dwell at inphase. In the human trial, ϕ_{34} decreased for almost one cycle before it stopped at inphase (0-10s yellow line in Fig 6A). This is not the case with constant frequency (Fig 6B, yellow line, ϕ_{34} immediately increases to inphase after the beginning of the trial), but it is the case with varying frequency (Fig S9B, yellow line, 0-5s). All these show by a very simple example how gradual adaptation in natural frequency may cause sudden changes in coordination patterns.

A note on metastability

For intuition, let us assume that there are N oscillators in a stationary organization defined by $N-1$ relative phases, each of which remains near inphase, near antiphase, or wrapping, giving us $S = 3^{N-1}$ different stationary patterns for our model ($S = 2^{N-1}$ for the Kuramoto model because of the lack of antiphase). Now if we look at patterns as sequences of metastable dwells, we could have $M = \sum_{l=1}^{S} \frac{S!}{(S-l)!}$ patterns of various period l (with non-repeated spatial configurations in sequence). These of course are not all necessarily reachable by a system, which in itself is an interesting theoretical problem, but still the repertoire M is much greater than S. This thought experiment shows how metastability contributes to biological complexity

in a very significant way.

Design of the human experiment

The human experiment (24) was performed by a total of 120 subjects in 15 ensembles. Each ensemble completed 18 trials (6 trials for each condition $\delta f = 0$, 0.3 and 0.6 Hz) of interaction in a complete network, except one ensemble for which only 7 trials (2 for $\delta f = 0$ Hz, 2 for $\delta f = 0.3$ Hz, and 3 for $\delta f = 0.6$ Hz) were completed due to equipment malfunction. This yields 86 trials for $\delta f = 0$ Hz, 86 trials for $\delta f = 0.3$ Hz, and 87 trials for $\delta f = 0.6$ Hz. See (24) for additional details.
